# Crisis Migration Adverse Childhood Events: A New Category of Youth Adversity for Crisis Migrant Children and Adolescents

**DOI:** 10.1007/s10802-022-01016-x

**Published:** 2023-01-10

**Authors:** Beyhan Ertanir, Cory L. Cobb, Jennifer B. Unger, Teresa Celada-Dalton, Amy E. West, Ingrid Zeledon, Patrizia A. Perazzo, Miguel Ángel Cano, Sabrina E. Des Rosiers, Maria C. Duque, Simon Ozer, Natalie Cruz, Carolina Scaramutti, Saskia R. Vos, Christopher P. Salas-Wright, Mildred M. Maldonado-Molina, Lea Nehme, Charles R. Martinez, Luis H. Zayas, Seth J. Schwartz

**Affiliations:** 1 School of Education, Institute Research and Development, University of Applied Sciences and Arts Northwestern Switzerland, Bahnhofstrasse 6, 5210 Windisch, Switzerland; 2 Auburn University, Auburn, United States; 3 University of Southern California, Los Angeles, United States; 4 Pepperdine University, Malibu, United States; 5 University of Texas at Austin, Austin, United States; 6 Florida International University, Miami, United States; 7 Barry University, Miami, United States; 8 Aarhus University, Aarhus, Denmark; 9 Children’s Hospital Los Angeles, Los Angeles, United States; 10 University of Miami (Florida), Miami, United States; 11 Boston College, Chestnut Hill, United States; 12 University of Florida, Gainesville, United States

**Keywords:** Immigration, Adverse childhood events, Crisis migration, Trauma

## Abstract

The present article proposes an extension of the concept of adverse childhood experiences (ACEs) to apply to crisis migration – where youth and families are fleeing armed conflicts, natural disasters, community violence, government repression, and other large-scale emergencies. We propose that adverse events occurring prior to, during, and following migration can be classified as crisis-migration-related ACEs, and that the developmental logic underlying ACEs can be extended to the new class of crisis-migration-related ACEs. Specifically, greater numbers, severity, and chronicity of crisis-migration-related ACEs would be expected to predict greater impairments in mental and physical health, poorer interpersonal relationships, and less job stability later on. We propose a research agenda centered around definitional clarity, rigorous measurement development, prospective longitudinal studies to establish predictive validity, and collaborations among researchers, practitioners, and policymakers.

## Crisis Migration Adverse Childhood Events: A New Category of Youth Adversity for Immigrant Children and Adolescents

Adverse childhood events (ACEs), defined as “experiences which require significant adaptation by the developing child in terms of psychological, social and neurodevelopmental systems, and which are outside of the normal expected environment” (Lacey & Minnis, 2020, p. 116; McLaughlin 2016), have been commonly studied as social conditions and occurrences that predispose youth toward subsequent poorer health and psychosocial outcomes (Chapman et al., 2007). ACEs include familial or household conditions such as parental divorce, having a parent in jail, repeatedly witnessing family violence, and experiencing child abuse or neglect (Felitti et al., 1998; Finkelhor et al., 2013). The number, severity, and chronicity (i.e., how many, how impactful, and how long-lasting) of ACEs experienced during childhood and adolescence have been found to predict substance use disorders (Leza et al., 2021), obesity (Wiss et al., 2020), major depression (Lu et al., 2008), and an array of chronic health conditions (Hughes et al., 2017). Therefore, ACEs represent a powerful construct that captures early life adversity that has important implications for longer-term health trajectories.

The majority of the original ACE criteria (e.g., experiencing or not experiencing the 10 defined ACE types occurring in the household) apply to individuals from both native-born and immigrant backgrounds, and ACEs have been studied among both foreign-born and native-born individuals (e.g., Vaughn et al., 2017). Vaughn et al. found that United States (U.S.) native-born individuals reported significantly greater numbers of specific types of ACEs, such as experiences of physical and sexual abuse and witnessing domestic violence, compared to first-generation international migrants. This finding appears to suggest that migrants are likely to experience fewer adverse events than individuals born in the United States – and this finding comports with the larger literature on the “immigrant paradox” (see Alcántara et al., 2017, for a review), where migrants report more favorable mental and physical health compared to U.S.-born individuals. Similarly, Caballero et al., (2017) found that, despite a higher prevalence of poverty, children from Hispanic immigrant families had a significantly lower prevalence of ACE exposure compared to children from U.S.-born families.

This pattern of differences between immigrants and native-born youth suggests some degree of health advantage for immigrants. Potential determinants of this disparity include factors such as community and family contexts, cultural values, and character strengths, all of which can play a crucial role in explaining the more favorable psychological health of (particularly first-generation) immigrants (Cobb et al., 2019). For example, among immigrants, studies have suggested that familial factors such as strong family ties, support, and cohesion (e.g., Falicov 2007, 2013; Parra-Cardona et al., 2006) or community networks can act as important resources for dealing or coping with adversity and for building resilience (Hull et al., 2008; Linton et al., 2016).

However, lower levels of ACEs among immigrants, relative to native-born individuals, may also be due, at least in part, to the lack of attention to specific adverse events that migrants experience (Cabarello et al., 2017; Conway & Lewin, 2022). More recently, scholars (McEwen & Gregorson, 2019) have proposed that currently available ACE theories and measurement tools do not consider specific types of adverse experiences that are specific to people from diverse populations. Some authors (Flores & Salazar, 2017; Zetino et al., 2020) note that the original ACE tool may not capture adverse experiences that are specific to immigrants and immigrant families.

Indeed, a study using the recently developed 13-item measure of “Immigrant-Specific Adverse Childhood Experiences” (ACE-I; Conway & Lewin 2022) found that many Hispanic migrant youth reported adversities on the ACE-I measure that were not included in the traditional ACE measure. Moreover, the National Child Traumatic Stress Network (NCTSN; Amaya-Jackson et al., 2021) noted at least 22 adverse experiences that are currently not captured by the original ACE items. Several of these experiences (e.g., war/terrorism/political violence, natural disasters, forced displacement) represent conditions that many migrant youth have confronted. Additionally, a recently published and more comprehensive, but non-migrant-specific, instrument *Adverse Life Experiences Scale* (Hawes et al., 2021), includes items related to discrimination, separation from one’s heritage culture or community, and exposure to war and conflict. The theory underlying this instrument emphasizes the potential for these specific experiences to predict developmental outcomes in similar ways as traditional ACEs do (Hawes et al., 2021). This and other conceptual work (see the section entitled Immigration-Related Adverse Childhood Events for Crisis Migrant Youth, below) suggest that adverse events occurring in migrant youth’s sending countries – and during the journey between the sending and destination countries, such as separation from family members and one’s culture of origin, fear of death or serious injury during the migration journey, and being detained at the border – should also be encompassed within the ACEs framework.

Thus, our approach in the present article builds on existing research (e.g., Barajas-Gonzalez et al., 2021; Conway & Lewin, 2022), but extends this work to apply specifically to crisis migration, which refers to refugees, asylum seekers, and other individuals and families leaving in large numbers because of natural or human disasters that create unlivable or unsustainable conditions in their home countries (Vos et al., 2021). Crisis migration includes, but is not synonymous with, refugees and asylum seekers – crisis migration also includes individuals who flee natural or human disasters, but who are not classified as refugees and who do not seek asylum.

Although the development of the ACE-I instrument (Conway & Lewin, 2022) represents an important first step in conceptualizing and assessing migration-related ACEs, we argue that several important issues remain unaddressed within the current migration-related ACE literature (e.g., Barajas-Gonzalez et al., 2021). These issues include (a) applying migration-related ACEs specifically to crisis migration, (b) developing crisis and context-specific comprehensive measures which meet the criteria of psychometric quality, (c) examining the overlap between normative and migration-related ACEs, and (d) adopting the developmental psychopathology perspective that underlies the original ACE framework. Currently, no ACE-related instrument addresses common crisis migrant experiences such as abrupt separation from family members, being cut off from one’s heritage culture, or hazardous post-migration living conditions (e.g., living in crowded and unsafe surroundings). Moreover, we also do not know whether newly created instruments, such as the ACE-I, are reliable and generate the same measurement properties across sub-populations of crisis migrants (e.g., individuals from different countries, accompanied versus unaccompanied minors). Further, while existing conceptual work (e.g., Barajas-Gonzalez et al., 2021; Caballero et al., 2017) is extremely valuable in advancing our understanding of crisis-migration related ACEs, this work is largely descriptive. As a result, further in-depth investigations of crisis migrants’ ACE-related experiences is needed.

Accordingly, the purpose of the present article is not only to expand the concept of ACEs to include pre-migration, transit-related, and post-migration adverse events, but also to propose such an expansion specifically for crisis migrants. Because of the adversities they experience, crisis migrant youth may be especially at risk for migration-specific ACEs (Vos et al., 2021). Conceptualizing adverse crisis-migration-related events as ACEs would bring the developmental psychopathology logic underlying the ACE construct to bear on crisis-migration-related adversities and would raise new research questions vis-à-vis how crisis-migration-related ACEs might interface with other types of ACEs to predict mental and behavioral health symptoms and disorders as developmental outcomes among crisis migrant youth.

This article is divided into three primary sections. First, we define and review literature on crisis migration, and we discuss the types of adverse events that many crisis-migrant youth experience. Second, we propose the concept of crisis-migration-related ACEs among crisis-migrant youth and delineate among different types of crisis-migration-related ACEs. Finally, we suggest future directions for research.

### Crisis Migrant Youth

Several types of international migrants have been proposed, including voluntary immigrants, refugees, and asylum seekers (Berry, 2017). Voluntary immigrants reach an intentional, often deliberate decision to relocate to a new country for reasons including employment opportunities, family reunification, or improved educational prospects for oneself or one’s children. Refugees and asylum seekers are often displaced by wars, natural disasters, or other uncontrollable events, and are either involuntarily resettled in a new country or seek permission to enter that country (United Nations High Commissioner for Refugees [UNHCR], 2021, 2022a).

Vos et al., (2021) have introduced an overarching category, *crisis migration*, which is more expansive and encompassing than the refugee and asylum seeker categories. Specifically, crisis migrants flee civil wars, natural disasters, dictatorial or repressive governments, and other emergencies and seek refuge in whatever countries will accept them. In many cases, crisis migration involves long treks through perilous territories or crossing large or dangerous bodies of water. Further, many crisis migrants are not refugees and do not seek asylum – they may enter the destination country without authorization or may overstay a visa. Crisis migrations are, by definition, large-scale out-migrations in which large numbers of individuals and/or families relocate in response to broad macro-level challenges (Vos et al., 2021). In many cases, crisis migrants include unaccompanied minors who are sent to escape the home country’s crisis when parents/guardians do not have the resources to relocate with the child (Menjívar & Perreira, 2019; Nardone & Correa-Velez, 2015).

According to the Family Crisis Migration Stress Framework (Vos et al., 2021), crisis migration is characterized by four primary factors. These are (a) sudden onset or occurrence (time factor), (b) a clear natural or human disaster (or set of disasters) that has prompted the migration (macro-level factor), (c) the large-scale flow of migrants to a number of receiving contexts (quantity factor), and (d) the compounded vulnerability (e.g., witnessing deadly violence, fearing one’s imminent death) that crisis migration imposes on migrants (effect factor). At a more granular level, crisis migration is largely an “overnight” migration to receiving contexts that are often not expecting or prepared to receive the migrants (Salas-Wright et al., 2022). Indeed, the term *crisis migrant* reflects the sudden and massive character of the migration wave (Martin et al., 2014).

One of the most notable crisis migrations in recent history is the Russia-Ukraine war, which has forced more than 7 million people to flee their homes to seek safety, protection, and assistance (UNHCR, 2022b). Other examples of recent crisis migrant waves – both of which are still ongoing – include the Syrian civil war (Buonanno, 2017) and the Venezuelan governmental and societal collapse (which sent millions of people fleeing to the United States and other Latin American countries; Wolfe, 2021). The large-scale movement of people – including many unaccompanied children – out of Central America’s “Northern Triangle of Violence” during the 2010s was another important example of crisis migration. However, although most of these examples involved crisis migrants relocating from one country to another, crisis migration can also include movements within the same country. For example, Hurricane Katrina, the largest and deadliest U.S. hurricane on record, caused one of the largest and most abrupt relocations of people in U. S. history (Geaghan, 2011). More than 1.5 million people were evacuated from their homes, with several thousand forced to move to other states for several months to years, and with many relocating permanently (Groen & Polivka, 2008).

Notably, all these crisis-driven migrations had an identifiable “tipping point” in which mass migration began or accelerated. The tipping point is defined by the growth of a country or region’s net emigration occurring over a shortened time frame due to events occurring in that country’s or region’s greater sociopolitical context (McAdam, 2014). The net migration rate (total number of immigrants less than the total number of emigrants; The World Bank Group, 2022) experiences a substantial increase over a relatively short period of time (e.g., several months to two to three years) which can be attributed to one event or series of events in the home country that render everyday life there untenable. Such tipping points are best understood as pivotal events that take place within a broader context of economic, social, political, or environmental instability (McAdam, 2014). As an example, Hurricane Maria in Puerto Rico prompted a large-scale out-migration from the U.S. island territory, but this migration must be understood within the broader context of declining infrastructure, economic stagnation, and social problems that predated the Category 4 hurricane (Salas-Wright et al., 2021). It should be noted that as climate-related severe weather and conditions intensify (e.g., more and stronger hurricanes, more severe droughts), crisis migration is expected to increase – and as a result, more and more individuals will be exposed to crisis-migration-related ACEs (Abel et al., 2019; Salas-Wright et al., 2021).

Work with child and adolescent migrants has focused primarily on unaccompanied minors – and for good reason. Unaccompanied minors, compared to youth who migrate with family members, may be more likely to witness or experience adverse events such as beatings, rape, and murder, more likely to have fled wars or natural disasters, and more likely to experience life-threatening medical problems (Bean et al., 2007; Rodriguez et al., 2019). Unaccompanied minors are often fleeing the direst of circumstances and often come from families where limited resources do not allow the whole family to migrate together. Indeed, when parents must make the difficult decision to send their youth on perilous journeys alone, this separation alone represents an important ACE.

Research with immigrants suggests that separation from family, culture, and community serves as an important source of stress (Sternberg et al., 2016). According to Self-Categorization Theory (Turner et al., 1987), through the sense of affiliation to one specific group or culture (and the mental and affective meaning of this affiliation), individuals understand their place in the world (Mummendey et al., 2009; Turner et al., 1987). So, group identification is central to an individual’s sense of belongingness and meaning of life. When immigrants leave their community and/or country, they often experience loss of culture, social ties, and social support as consequence of their separation from their social group. In most cases, this means also losing the source of behavioral repertoires that normally helps people to solve their daily problems. Such feelings of loss can be painful and may have consequences on their well-being and adaptation (Cobb et al., 2019).

Typically, during the three primary stages of migration (i.e., pre-migration, during migration, and post-migration), youth are often accompanied by their parents and/or family members (Bámaca-Colbert et al., 2018), who function as role models in terms of dealing and coping with challenges and adversities in their new lives (Ingoldsby & Smith, 1995). Family and community members are generally the main transmitters of the heritage culture and identity, which is of primary importance vis-à-vis immigrants’ psychological and physical health (Cariello et al., 2020; Yoon et al., 2020). Indeed, certain collectivist cultural values such as interdependence (Triandis, 2001), cohesion and loyalty (Bacallao & Smokowski, 2007), social connectedness (Yoon et al., 2008), and social harmony (Xie et al., 2004) have been shown to buffer risks of stress related to acculturation and adaptation (Cobb et al., 2019). These values are central to collectivist cultural streams and are essential to preserve for immigrants who move from primarily collectivist to primarily individualist cultural contexts (Motti-Stefanidi, 2018). Unaccompanied youth, however, do not have access to these familial resources. In cases where unaccompanied youth migrate from primarily collectivist to primarily individualist cultural contexts – such as Central Americans migrating to the United States or Syrians migrating to Germany – the absence of collectivist cultural values in the new national context may represent a considerable challenge. This challenge may be exacerbated by the absence of close family members who can serve as sources of support (see Garcia & Birman 2022, for a review). Thus, it seems reasonable to conclude that unaccompanied children might particularly suffer from yearning for their home country and culture; separation from family, peers, and familiar surroundings; and decreased social support and isolation. All of these experiences may qualify as ACEs.

Unaccompanied minors also face a greater likelihood of additional crisis-migration-related ACEs, such as exploitation, victimization, and a higher likelihood of mistreatment by border officials, compared to youth who migrate with family (Coulter et al., 2020; Rodriguez et al., 2019). “Tough” immigration policies leading to apprehension, detention, and deportation of unaccompanied minors are also likely to lead to adverse experiences or even traumatic interactions with immigration courts, border patrol agents, and immigration enforcement personnel (Pierce, 2015). Unaccompanied minors are often placed into foster care if a suitable guardian cannot be identified (Crea et al., 2018) and tend to be overrepresented among severely psychiatrically impaired youth (Ramel et al., 2015). Schools (Davila et al., 2020) and mental health services (Perreira & Pedroza, 2019) are often unprepared to address these experiences and their mental health sequelae.

Although unaccompanied minors are likely at the greatest risk for adverse experiences before, during, and following migration, youth who migrate with their parents are not immune to such adverse experiences. In the United States, for example, undocumented parents crossing the southern border have been detained separately from their children, with the children often moved to locations hours away from their parents (Linton et al., 2017). As of December 2020, hundreds of these children were still separated from their parents (Gonzalez, 2020). Empirical evidence suggests that these forced parent-child separations may lead to post-traumatic stress, anxiety, depression, and suicidal ideation among youth (Giano et al., 2020; Teicher, 2018). Many undocumented children and adolescents – including those protected in the United States by Deferred Action for Childhood Arrivals (DACA) – may experience severe and chronic anxiety stemming from the potential detention or deportation of their parents (Yoshikawa et al., 2017) and from the fear of DACA being ended. Further, undocumented youth – whether they migrate alone or with family members – are disproportionately likely to settle in poor, high-crime areas in their destination countries (Asad & Rosen, 2019). In turn, these neighborhood conditions are likely to predispose youth to additional adverse events.

Although not all undocumented immigrants are crisis migrants, many crisis migrants are undocumented or apply for asylum (Vos et al., 2021; Chand et al., 2017) analyzed asylum decisions rendered by U.S. immigration judges and found that Central Americans – who represented approximately 25% of all asylum requests filed between January 2017 and January 2019 (United States Customs and Immigration Services, 2019) – were significantly less likely to be granted asylum compared to petitioners from other countries or regions. Similarly, many European countries have taken steps to prevent Syrians – who are one of the largest groups of prospective asylum seekers in Europe – from applying for asylum (Karageorgiou, 2016). Therefore, individuals who apply for asylum, or who intend to do so, may become undocumented if their asylum petitions are rejected or if their destination countries refuse to consider their asylum requests. Among immigrant youth, unauthorized status has been linked with unfavorable economic and educational opportunities and aspirations, poor sense of belonging, stigma, and poor mental and emotional well-being (Castañeda, 2019; Gonzales, 2011; Gonzales et al., 2013), all of which represent risk factors for unfavorable life outcomes.

### Immigration-Related Adverse Childhood Events for Crisis Migrant Youth

We propose that crisis-migration-related adverse events that occur during childhood and adolescence should be categorized under the heading of ACEs – specifically as crisis-migration-related ACEs. Such classification is not only a useful empirical and definitional exercise, but it also provides mechanisms and opportunities through which youth in need of clinical care can be identified and referred (see Jones et al., 2020, for further discussion).

Similar arguments and ideas have been advanced in other conceptual works. Barajas-Gonzalez et al., (2021) introduced the *Immigration-Related Adverse Childhood Experiences Model*, which proposes that immigration-related threats and deprivation should be included within the ACE framework. More specifically, Barajas-Gonzalez et al. argue that experiences and threats of detention and deportation (either their own or of a family member), with which many children from immigrant families are faced, should be considered as ACEs – as should the systematic marginalization and deprivation that many immigrants experience.

Following Vos et al., (2021), Conway & Lewin (2022), and others, these ACEs might be subdivided into those occurring prior to, during, and following migration. Exposure to wars, natural disasters, gang violence, or government repression in one’s country of origin would represent pre-migration ACEs; being raped, beaten, or threatened with death (either by other people or by accidents such as vehicle crashes or boats capsizing) would represent examples of in-transit ACEs; and being held in a detention center or fearing deportation would represent examples of post-migration ACEs. The cumulative logic underlying ACEs – where greater severity and chronicity of ACEs predict worse mental and physical health outcomes (Leza et al., 2021; Wiss et al., 2020) – would be applied to crisis-migration-related ACEs as well, such that greater numbers of crisis-migration-related ACEs – especially ACEs that are particularly chronic and/or severe – would be expected to predict more severe mental and physical impairments and disease (Kliethermes et al., 2014), and likely impaired flourishing and well-being.

Some work in this direction has been conducted, but more research is still needed. Flores and Salazar (2017, p. 2) argue that “(1) U.S. Immigration and Customs Enforcement (ICE) arrests or deportations of parents or guardians, (2) being a victim of or witnessing ICE arrests or raids, (3) parent or guardian separation because of migration, and (4) experiencing anti-immigrant discrimination” should be integrated into ACE measures, but they do not suggest any specific items that should be used to measure these experiences. Moreover, an Australian research group (Hanes et al., 2017) added several refugee-specific ACE items to a standard ACE measure. These refugee-specific items included migration journeys lasting longer than five years, current or prior separations from nuclear family members, being detained, and witnessing traumatic events. Their study, however, was primarily descriptive, and they did not examine the extent to which refugee-specific ACEs contributed (either alone or in concert with other ACEs) to mental or physical health symptoms among the refugee youth in their sample. Lastly, the ACE-I (Conway & Lewin, 2022) captures a wide range of migration-related adversities. However, in terms of methodological aspects and psychometric properties, questions remain regarding this and other potential immigration-related ACE measures (for a more detailed discussion, see the Rigorous Measurement Development section below) – and the overlap between, and combined predictive power of, traditional and crisis-migration-related ACEs has yet to be examined.

### Unanswered Research Questions on Crisis Migration ACEs

The expansion of the ACEs concept to apply to crisis migration raises several important research questions. These include (a) What are the developmental pathways linking crisis-migration-related ACEs to longer-term mental health outcomes? (b) How do various crisis-migration-related ACEs relate to developmental and mental health outcomes of interest? That is, which crisis-migration-related ACEs are most proximal to youth development and risk, and how do crisis-migration-related ACEs interact with, or compound, other ACEs and forms of adversity to influence youth development and mental health? (c) What items and events best capture ACEs among crisis migrant youth? How do these items function and/or vary across subsets of crisis migrant youth (e.g., biological sex, younger vs. older youth)? How do they relate to outcomes of interest? (d) What are the most appropriate strategies for developing culturally sensitive measures for crisis-migrant ACEs? What are the most important design considerations for moving forward?

Here, it is important to note that all of these questions also apply to migration-related ACEs as introduced by Conway and Lewin (2022). Thus, we propose that research on crisis-migration-related and other migration-related ACEs should be integrated with the service of understanding and intervening in the lives of crisis migrant youth.

To address these unanswered research questions, we suggest three primary steps needed to launch a line of research on crisis-migration-related ACEs. These steps involve (a) integrating crisis-migration-related ACEs with broader understandings of child and adolescent development, (b) rigorous measurement of crisis-migration-related ACEs, and (c) using a combination of emic and etic (rather than solely etic) methods to identify the crisis-migration-related ACEs to be measured and studied. We briefly discuss each of these steps below.

### Integrating Migration-Related ACEs within Broader Understandings of Child and Adolescent Development

In this section, we propose a developmental perspective on crisis-migration ACEs and their effects on subsequent life outcomes among child and adolescent crisis migrants.

From a developmental psychopathology perspective (Cicchetti & Rogosch, 2002), there are myriad pathways through which early childhood adversity may exert its effects on mental health outcomes, and individuals vary in the degree to which they are affected by such adversity. Whereas there are many pathways that may result in a poor outcome for certain youth (equifinality), depending on a host of factors, other youth may be more or less affected by the same pathways (multifinality; Cicchetti & Rogosch 1996). In the context of ACEs, early childhood adversities represent multiple forms of stress that can result in poor mental health later in life (Nurius et al., 2016). Such adversities may exert their effects on health through multiple biological and social pathways, including biological changes that disrupt maturational and stress-response systems (Danese & McEwen, 2012); increased vulnerability to illness through inequality (poverty, poor living conditions, greater risk of social difficulties; Nurius et al., 2016); and reductions in psychosocial resources such as friends, family, and other forms of social support (Vranceanu et al., 2007). However, the ways in which these pathways will affect immigrant youth will depend largely upon their available coping resources and abilities (Hill et al., 2010). To advance the study of crisis-migration ACEs, it is critical that we identify the specific pathways through which childhood adversity results in poor outcomes later in life, as well as the culturally-specific protective factors that can buffer against these negative effects.

Crisis-migrant youth not only experience adversities before, during, and following migration, but they also function within the normative social contexts of childhood and adolescence – such as family, peers, school, and neighborhood (Noack, 2021). These youth pass through puberty, experience normative (as well as adversity-influenced) family relationships, may experience discrimination and other culturally related stressors, and are tasked with succeeding in school and establishing friendships with similarly aged peers (Juang et al., 2018; Suárez-Orozco et al., 2018). Schools are essential contexts for adolescent development – youth not only learn class material, but also acquire critical social and self-regulation skills from interacting with peers and teachers (Eccles & Roeser, 2011; Steinberg & Morris, 2001). In addition to the adverse events that they may have experienced prior to and during migration, many crisis-migrant youth must attend school in a new language that they may not speak or understand well (Sirin & Rogers-Sirin, 2015). Moreover, they also must navigate between different sets of cultural values and norms at school, and these potential cultural conflicts at school may serve as an additional stressor (Motti-Stefanidi & Masten, 2017). As a result, schools, peers, and teachers can serve as settings that facilitate normative healthy development, as well as potentially functioning as additional risk factors (Motti-Stefanidi, 2018).

Further, a developmental lens suggests that youth can be oriented toward resilience as well as toward pathology (Southwick et al., 2014). Even the most traumatized individuals nonetheless possess important strengths, and these strengths can be capitalized on to promote positive developmental outcomes (e.g., school success, close friendships, warm and trusting family relationships). For example, Keles et al., (2018) found that, among a sample of unaccompanied minors in Norway, heritage culture maintenance differentiated those youth whose depressive symptoms were consistently low across two timepoints from youth who reported more symptoms of depression at either timepoint. This finding might be used, for example, to design interventions to encourage these youth to retain their heritage culture as a way of promoting resilience against mental health problems. It would also be important to examine whether heritage-culture maintenance helps to offset the effects of migration-related ACEs on subsequent internalizing symptoms, as is the case among voluntary immigrant youth (Cobb et al., 2019).

A developmental perspective also suggests that crisis-migration-related ACEs might add to, or interact with, other ACEs (Amaya-Jackson et al., 2021) to predict outcomes such as life satisfaction, peer relationships, school success, anxiety, or depressive symptoms. An amplification hypothesis, for example, might suggest that experiencing child abuse (an ACE that is not directly related to migration) in the destination country or region might exacerbate the predictive effects of separation from parents (a crisis-migration-related ACE) on developmental outcomes. In contrast, a buffering hypothesis might suggest that fearing one’s own deportation, or that of a family member (a crisis-migration-related ACE), would dilute the predictive effects of witnessing domestic violence in the destination country or region (an ACE that is not necessarily related to migration) on developmental outcomes. Hypotheses such as these reflect the embeddedness of crisis-migrant youth within the developmental realities of childhood and adolescence as well as within the cultural realities of crisis migration and its sequelae. Nevertheless, to explore this “terra incognita,” rigorous measurement is crucial. We now turn to a discussion of this topic.

### Rigorous Measurement Development

Considerable measurement development work has been conducted with special populations such as refugees and other migrant groups. The process of developing psychometrically rigorous measures is labor-intensive and requires a great deal of care (Leong et al., 2010). Steps within this process include conducting qualitative research with the target group(s) to define the problem, assembling an expert panel to help develop items, conducting additional qualitative research (including convening focus groups) to review these items and generate new items, collecting data to examine the psychometric properties (e.g., reliability, validity, objectivity) of these items and its appropriateness for other contexts and groups. Thus far, no published work has completely followed these steps to develop measures for crisis-migration-related ACEs. Even though Conway and Lewin’s (2022) measure appears promising, their proposed items were selected from existing current migration stress literature (for examples, see Mohamed & Thomas 2017; Perreira & Ornelas, 2013) rather than specifically developed for the purpose of assessing migration-related ACEs. Moreover, measurement invariance across gender, age, or national origin are not known, and reliability and validity information has not been reported.

In addition to the ACE-I (Conway & Lewin, 2022), Hanes et al., (2017) have proposed ACE items for refugees; and Hawes et al. (2021) have introduced items related to discrimination, separation from culture or community, and exposure to war and conflict. These scales have facilitated progress toward closing some gaps concerning immigration-related ACEs. Moreover, other scales, such as the Structured Trauma-Related Experiences and Symptoms Screener (Grasso et al., 2015) and the War Trauma Screening Scale (Layne et al., 1999), might be also used to assess adverse experiences among crisis-migrant youth.

However, existing measures may be characterized by key limitations. First, the Structured Trauma-Related Experiences and Symptoms Screener and the War Trauma Screening Scale are primarily trauma-focused and do not appear to fully capture the heterogeneity of crisis-migrant youth’s adverse experiences (Amaya-Jackson et al., 2021). Trauma and ACEs are not interchangeable, and not all adverse events will result in trauma. Second, the utility of the Conway, Hanes, and Hawes measures may also carry some limitations vis-à-vis crisis-migration-related ACEs, as other ACEs may occur within specific contexts (thereby requiring at least some context-specific measurement). These context-specific ACEs may differ not only in terms of geographic or cultural aspects, but also in terms of the types of crises that need to be measured.

Thus, we recommend that scholars continue to conduct research in this direction, using existing measures as a guide, to study crisis migration ACEs further. We highly encourage scholars to conduct focus groups and individual interviews to supplement existing measures with additional context-specific items.

### Using Combined Emic-Etic Methods to Identify Crisis-Migration-Related ACEs

In cross-cultural psychology, etic approaches (i.e., those rooted in outsider perspectives) carry the risk of imposing norms and values from the researcher’s own (generally Western) perspective and context, whereas emic approaches (i.e., from insider perspectives) draw on indigenous wisdom from the group being studied (see Leong et al., 2010, for a review). Using only etic methods can ignore or distort key aspects of the phenomenon under study. For example, in a systematic review of studies of grief among refugees and post-conflict survivors (Killikelly et al., 2018), studies using emic approaches, or a combination of emic and etic approaches, found that up to 76% of participants reported maladaptive grieving (e.g., preoccupation with deceased loved ones). In contrast, studies using only standardized, etic scales found that only 32% of participants reported maladaptive grieving. A key conclusion is that standardized etic scales overlooked (or otherwise missed) important dimensions of maladaptive grieving.

Leong et al., (2010) suggest using a combined emic-etic strategy, where the rigor of standardized scales is integrated with first-person accounts from members of the target ethnic or cultural group. To our knowledge, except for the ACE-I developed by Conway and Lewin (2022), all other existing scales and measures appear to have been developed using an etic rather than emic process. Considering the value of emic and combined emic-etic approaches, we propose that items such as those developed by Conway and Lewin (2022) might be supplemented with additional items generated through focus groups and interviews with crisis-migrant youth, their family members, and practitioners and policymakers who serve this population. Researchers can identify some context- and crisis-specific adverse experiences that should be included within theories, measures, and research programs focusing on crisis-migration-related ACEs, but the youth themselves (as well as individuals connected to them) also must provide input. The development of the ACE-I measure (Conway & Lewin, 2022) followed such a procedure by including immigrant youth themselves, experts in the field, and service providers throughout the item selection process.

What we are suggesting, then, is a process through which existing measures, such as the ACE-I, can be supplemented with indigenous wisdom from (and experiences among) the specific migrant groups being studied – and through which new measures can be developed. Clearly, some experiences – such as separation from family members – are likely to occur regardless of the migrant group or destination country in question. However, other circumstances, such as stressors experienced prior to migration, specific adverse experiences occurring during the journey to the destination country, and cultural stressors encountered after entering the destination country, may differ across crisis-migrant groups. It may be wise to begin with a set of items that apply across crisis-migrant groups and use qualitative methods to generate additional items for the specific groups under study.

## Conclusion

In the present article, we have introduced the concept of crisis-migration-related adverse childhood events. These migration-specific ACEs may occur in addition to ACEs that are not specific to migration (e.g., child abuse, parental incarceration, being bullied). Casting adversities occurring in the lives of migrant youth before, during, and following migration as ACEs lends a developmental psychology and psychopathology perspective to phenomena that have generally not been studied using developmental theories or methods. Essential directions to follow in this line of research include developing and validating measures of crisis-migration-related ACEs, examining the effects of these ACEs (along with other types of ACEs) on later developmental outcomes, and identifying moderators and mediators that can help to offset the effects of crisis migration-related ACEs on outcomes. In turn, such steps may also facilitate the design, adaptation, and delivery of interventions and policies to improve the lives of crisis migrants in their new homelands. We hope that the present work helps to open a line of empirical and applied efforts in this direction.

## References

[CR1] Abel GJ, Bottrager M, Cuaresma JC, Muttarak J (2019). Climate, conflict, and forced migration. Global Environmental Change.

[CR2] Alcántara, C., Estevez, C. D., & Alegría, M. (2017). Latino and asian immigrant adult health: Paradoxes and explanations. In S. J. Schwartz, & J. B. Unger (Eds.), *Oxford Handbook of Acculturation and Health* (pp. 197–220). Oxford University Press.

[CR3] Amaya-Jackson, L., Absher, L. E., Gerrity, E. T., Layne, C. M., & Halladay Goldman, J. (2021). *Beyond the ACE score: perspectives from the NCTS on Child Trauma and Adversity Screening and Impact*. National Center for Child Traumatic Stress.

[CR4] Asad A, Rosen E (2019). Hiding within racial hierarchies: how undocumented immigrants make residential decisions in an american city. Journal of Ethnic and Migration Studies.

[CR5] Bacallao ML, Smokowski PR (2007). The costs of getting ahead: mexican family system changes after immigration. Family Relations.

[CR6] Bámaca-Colbert MY, Gonzales‐Backen M, Henry CS, Kim PS, Roblyer MZ, Plunkett SW, Sands T (2018). Family profiles of cohesion and parenting practices and latino youth adjustment. Family Process.

[CR7] Barajas-Gonzalez, R. G., Ayón, C., Brabeck, K., Rojas-Flores, L., & Valdez, C. R. (2021). An ecological expansion of the adverse childhood experiences (ACEs) framework to include threat and deprivation associated with U.S. immigration policies and enforcement practices: An examination of the Latinx immigrant experience. *Social Science & Medicine*, *282*, Article 114126. 10.1016/j.socscimed.2021.114126 10.1016/j.socscimed.2021.114126 PMC10409596 34146987

[CR8] Bean T, Derluyn I, Eurelings-Bontekoe E, Broeckart E, Spinhoven P (2007). Comparing psychological distress, traumatic stress reactions, and experiences of unaccompanied refugee minors with experiences of adolescents accompanied by parents. Journal of Nervous and Mental Disease.

[CR9] Berry, J. W. (2017). Theories and models of acculturation. In S. J. Schwartz, & J. B. Unger (Eds.), *Oxford handbook of acculturation and health* (pp. 15–28). Oxford University Press.

[CR10] Buonanno, L. (2017). The european migration crisis. In D. Dinan, N. Nugent, & W. E. Patterson (Eds.), *The European Union in crisis* (pp. 100–130). Palgrave Macmillan.

[CR11] Caballero, T. M., Johnson, S. B., Buchanan, C. R. M., & DeCamp, L. R. (2017). Adverse childhood experiences among hispanic children in immigrant families versus US-native families. *Pediatrics*, *140*(5), 10.1542/peds.2017-0297. Article e20170297. 10.1542/peds.2017-0297 28993445

[CR12] Cariello AN, Perrin PB, Williams CD, Espinoza GA, Morlett-Paredes A, Moreno OA, Trujillo MA (2020). Moderating influence of enculturation on the relations between minority stressors and physical health via anxiety in latinx immigrants. Cultural Diversity and Ethnic Minority Psychology.

[CR13] Castañeda, H. (2019). *Borders of belonging: struggle and solidarity in mixed-status immigrant families*. Stanford University Press.

[CR14] Chand DE, Schreckhise WD, Bowers ML (2017). The dynamics of state and local contexts and immigration asylum hearing decisions. Journal of Public Administration Research and Theory.

[CR15] Chapman DP, Dube SR, Anda RF (2007). Adverse childhood events as risk factors for negative mental health outcomes. Pediatric Annals.

[CR16] Cicchetti D, Rogosch FA (1996). Equifinality and multifinality in developmental psychopathology. Development and Psychopathology.

[CR17] Cicchetti D, Rogosch FA (2002). A developmental psychopathology perspective on adolescence. Journal of Consulting and Clinical Psychology.

[CR18] Cobb CL, Branscombe NR, Meca A, Schwartz SJ, Xie D, Zea MC, Molina LE, Martinez CR (2019). Toward a positive psychology of immigrants. Perspectives on Psychological Science.

[CR19] Conway, C. A., & Lewin, A. (2022). Development and Psychometric Properties of the ACE-I: Measuring Adverse Childhood Experiences Among Latino Immigrant Youth. Psychological Trauma: Theory, Research, Practice, and Policy. Advance online publication. 10.1037/tra0001223 10.1037/tra0001223 35113626

[CR20] Coulter K, Sabo S, Martínez D, Chisholm K, Gonzalez K, Zavala SB, Villalobos E, Garcia D, Levy T, Slack J (2020). A study and analysis of the treatment of mexican unaccompanied minors by customs and border protection. Journal on Migration and Human Security.

[CR21] Crea TM, Lopez A, Hasson RG, Evans K, Palleschi C, Underwood D (2018). Unaccompanied immigrant children in long term foster care: identifying needs and best practices from a child welfare perspective. Children and Youth Services Review.

[CR22] Danese A, McEwen BS (2012). Adverse childhood experiences, allostasis, allostatic load, and age-related disease. Physiology & Behavior.

[CR23] Davila C, Hill D, McCormick AS, Villarreal AL (2020). Child immigration from both sides of the border: implications for trauma-informed practices in the schools. Contemporary School Psychology.

[CR24] Eccles JS, Roeser RW (2011). Schools as developmental contexts during adolescence. Journal of Research on Adolescence.

[CR25] Falicov CJ (2007). Working with transnational immigrants: expanding meanings of family, community, and culture. Family Process.

[CR26] Falicov, C. J. (2013). *Latino families in therapy* (2nd ed.). Guilford Press.

[CR27] Felitti VJ, Anda RF, Nordenberg D, Williamson DF, Spitz AM, Edwards V, Marks JS (1998). Relationship of childhood abuse and household dysfunction to many of the leading causes of death in adults: the adverse childhood experiences (ACE) study. American Journal of Preventive Medicine.

[CR28] Finkelhor D, Shattuck A, Turner H, Hamby S (2013). Improving the adverse childhood Experiences Scale. JAMA Pediatrics.

[CR29] Flores, G., & Salazar, J. C. (2017). Immigrant latino children and the limits of questionnaires in capturing adverse childhood events. *Pediatrics*, *140*(5), 10.1542/peds.2017-2842. Article e20172842. 10.1542/peds.2017-2842 28993444

[CR30] Garcia MF, Birman D (2022). Understanding the migration experience of unaccompanied youth: a review of the literature. American Journal of Orthopsychiatry.

[CR31] Geaghan (2011). Forced to Move: An Analysis of Hurricane Katrina Movers 2009 American Housing Survey: New Orleans: SEHSD Working Paper Number 2011-17. U.S. Census Bureau.

[CR32] Giano Z, Anderson M, Shreffler KM, Cox RB, Merten MJ, Gallus KL (2020). Immigration-related arrest, parental documentation status, and depressive symptoms among early adolescent Latinos. Cultural Diversity and Ethnic Minority Psychology.

[CR33] Gonzales RG (2011). Learning to be illegal: undocumented youth and shifting legal contexts in the transition to Adulthood. American Sociological Review.

[CR34] Gonzalez, D. (2020, December 11). *628 parents of separated children are still missing. Here’s why immigrant advocates can’t find them*. USA Today. https://www.usatoday.com/story/news/nation/2020/12/11/immigrant-advocates-cant-locate-parents-separated-border-children/3896940001/.

[CR35] Gonzales RG, Suárez-Orozco C, Dedios-Sanguineti MC (2013). No place to Belong: contextualizing concepts of Mental Health among undocumented immigrant youth in the United States. American Behavioral Scientist.

[CR36] Grasso DJ, Felton JW, Reid-Quiñones K (2015). The structured trauma-related experiences and symptoms screener (STRESS): development and preliminary psychometrics. Child Maltreatment.

[CR37] Groen JA, Polivka AE (2008). Hurricane Katrina evacuees: who they are, where they are, and how they are faring. Monthly Labor Review.

[CR38] Hanes G, Sung L, Mutch R, Cherian S (2017). Adversity and resilience amongst resettling western australian paediatric refugees. Journal of Paediatrics and Child Health.

[CR39] Hawes DJ, Lechowicz M, Roach A, Fisher C, Doyle FL, Noble S, Dadds MR (2021). Capturing the developmental timing of adverse childhood experiences: the adverse life experiences Scale. American Psychologist.

[CR40] Hill TD, Kaplan LM, French MT, Johnson RJ (2010). Victimization in early life and mental health in adulthood: an examination of the mediating and moderating influences of psychosocial resources. Journal of Health and Social Behavior.

[CR41] Hughes K, Bellis MA, Hardcastle KA, Sethi D, Butchart A, Mikton C, Jones L, Dunne MP (2017). The effect of multiple adverse childhood experiences on health: a systematic review and meta-analysis. Lancet Public Health.

[CR42] Hull P, Kilbourne B, Reece M, Husaini B (2008). Community involvement and adolescent mental health: moderating effects of race/ethnicity and neighborhood disadvantage. Journal of Community Psychology.

[CR43] Ingoldsby, B. B., & Smith, S. E. (1995). *Families in multicultural perspective*. Guilford Press.

[CR44] Jones CM, Merrick MT, Houry DE (2020). Identifying and preventing adverse childhood experiences: implications for clinical practice. Journal of the American Medical Association.

[CR103] Juang Linda P., Simpson Jeffry A., Lee Richard M., Rothman Alexander J., Titzmann Peter F., Schachner Maja K., Korn Lars, Heinemeier Dorothee, Betsch Cornelia (2018). Using attachment and relational perspectives to understand adaptation and resilience among immigrant and refugee youth.. American Psychologist.

[CR45] Karageorgiou E (2016). Solidarity and sharing in the common european asylum system: the case of syrian refugees. European Politics and Society.

[CR46] Keles S, Friborg O, Idsøe T, Sirin S, Oppedal B (2018). Resilience and acculturation among unaccompanied refugee minors. International Journal of Behavioral Development.

[CR47] Killikelly, C., Bauer, S., & Mercker, A. (2018). The assessment of grief in refugees and post-conflict survivors: A narrative review of etic and emic research. *Frontiers in Psychology, 9*,1957. 10.3389/fpsyg.2018.01957 10.3389/fpsyg.2018.01957 PMC6204364 30405474

[CR48] Kliethermes M, Schacht M, Drewry K (2014). Complex trauma. Child and Adolescent Psychiatric Clinics of North America.

[CR49] Lacey RE, Minnis H (2020). Practitioner review: twenty years of research with adverse childhood experience scores–advantages, disadvantages and applications to practice. Journal of Child Psychology and Psychiatry.

[CR50] Layne, C. M., Stuvland, R., Saltzman, W., Djapo, N., & Pynoos, R. S. (1999). War trauma screening scale. *Unpublished manuscript*.

[CR51] Leong FTL, Leung K, Cheung FM (2010). Integrating cross-cultural psychology research methods into ethnic minority psychology. Cultural Diversity and Ethnic Minority Psychology.

[CR52] Leza L, Siria S, López-Goñi JJ, Fernández-Montalvo J (2021). Adverse childhood experiences (ACEs) and substance use disorder (SUD): a scoping review. Drug and Alcohol Dependence.

[CR53] Linton, J., Griffin, M., M., & Shapiro, A. (2017). Detention of immigrant children. *Pediatrics*, *139*, 10.1542/peds.2017-0483. Article e20170483. 10.1542/peds.2017-0483 28289140

[CR54] Linton JM, Choi R, Mendoza F (2016). Caring for children in immigrant families: vulnerabilities, resilience, and opportunities. Pediatric Clinics.

[CR55] Lu W, Mueser KT, Rosenberg SD, Jankowski MK (2008). Correlates of adverse childhood experiences among adults with severe mood disorders. Psychiatric Services.

[CR56] Martin S, Weerasinghe S, Taylor A (2014). What is crisis migration?. Forced Migration Review.

[CR57] McAdam J (2014). The concept of crisis migration. Forced Migration Review.

[CR58] McEwen CA, Gregerson SF (2019). A critical assessment of the adverse childhood experiences study at 20 years. American Journal of Preventive Medicine.

[CR59] McLaughlin KA (2016). Future directions in childhood adversity and youth psychopathology. Journal of Clinical Child & Adolescent Psychology.

[CR60] Menjívar C, Perreira KM (2019). Undocumented and unaccompanied: children of migration in the European Union and the United States. Journal of Ethnic and Migration Studies.

[CR61] Mohamed S, Thomas M (2017). The mental health and psychological well-being of refugee children and young people: an exploration of risk, resilience and protective factors. Educational Psychology in Practice.

[CR62] Motti-Stefanidi F (2018). Resilience among immigrant youth: the role of culture, development and acculturation. Developmental Review.

[CR63] Motti-Stefanidi, F., & Masten, A. S. (2017). A resilience perspective on immigrant youth adaptation and development. In N. J. Cabrera, & B. Leyendecker (Eds.), *Handbook on positive development of Minority Children and Youth* (pp. 19–34). Springer.

[CR64] Mummendey, A., Kessler, T., & Otten, S. (2009). Sozialpsychologische Determinanten–Gruppenzugehörigkeit und soziale Kategorisierung. *Diskriminierung und Toleranz* (pp. 43–60). VS Verlag für Sozialwissenschaften. 10.1007/978-3-531-91621-7_2.

[CR65] Nardone M, Correa-Velez I (2015). Unpredictability, invisibility and vulnerability: unaccompanied asylum-seeking minors’ journeys to Australia. Journal of Refugee Studies.

[CR66] Noack P (2021). Migrant youths: typical aspects of development during the adolescent years, specific challenges of growing up somewhere else, and some things we need to understand better. New Directions for Child and Adolescent Development.

[CR67] Nurius PS, Green S, Logan-Greene P, Longhi D, Song C (2016). Stress pathways to health inequalities: embedding ACEs within social and behavioral contexts. International Public Health Journal.

[CR68] Parra-Cardona JR, Bulock LA, Imig DR, Villarruel FA, Gold SJ (2006). Trabajando duro todos los días”: learning from the life experiences of mexican-origin migrant families. Family Relations.

[CR69] Perreira KM, Ornelas I (2013). Painful passages: traumatic experiences and post-traumatic stress among U.S. immigrant latino adolescents and their primary caregivers. The International Migration Review.

[CR70] Perreira KM, Pedroza JM (2019). Policies of exclusion: implications for the health of immigrants and their children. Annual Review of Public Health.

[CR71] Pierce, S. (2015). *Unaccompanied child migrants in U.S. communities, immigration court, and schools*.Migration Policy Institute. https://www.migrationpolicy.org/sites/default/files/publications/UAC-Integration-FINALWEB.pdf.

[CR73] Ramel, B., Täljemark, J., Lindgren, A., & Johansson, B. A. (2015). Overrepresentation of unaccompanied refugee minors in inpatient psychiatric care. *SpringerPlus*, *4*, 10.1186/s40064-015-0902-1. Article 131. 10.1186/s40064-015-0902-1 PMC4372620 25825687

[CR74] Rodriguez N, Urrutia-Rojas X, Gonzalez LR (2019). Unaccompanied minors from the Northern Central American countries in the migrant stream: social differentials and institutional contexts. Journal of Ethnic and Migration Studies.

[CR75] Salas-Wright CP, Maldonado-Molina MM, Brown EC, Bates M, Rodríguez J, García MF, Schwartz SJ (2021). Cultural stress theory in the context of family crisis migration: implications for behavioral health with illustrations from the adelante boricua study. American Journal of Criminal Justice.

[CR76] Salas-Wright, Maldonado-Molina MM, Pérez-Gómez A, Trujillo JM, Schwartz SJ (2022). The venezuelan diaspora: Migration-related experiences and mental health. Current Opinion in Psychology.

[CR78] Sirin SR, Rogers-Sirin L (2015). The educational and mental health needs of syrian refugee children.

[CR79] Southwick, S. M., Bonanno, G. A., Masten, A. S., Panter-Brick, C., & Yehuda, R. (2014). Resilience definitions, theory, and challenges: interdisciplinary perspectives. Psychotraumatology, 5, Article 25338. 10.3402/ejpt.v5.25338 10.3402/ejpt.v5.25338 PMC4185134 25317257

[CR80] Steinberg L, Morris AS (2001). Adolescent development. Annual Review of Psychology.

[CR81] Sternberg RM, Nápoles AM, Gregorich S, Paul S, Lee KA, Stewart AL (2016). Development of the stress of Immigration Survey (SOIS): a field test among mexican immigrant women. Family & Community Health.

[CR82] Suárez-Orozco C, Motti-Stefanidi F, Marks A, Katsiaficas D (2018). An integrative risk and resilience model for understanding the adaptation of immigrant-origin children and youth. American Psychologist.

[CR83] Teicher, M. H. (2018). Childhood trauma and the enduring consequences of forcibly separating children from parents at the United States border. *BMC Medicine*, *16*, Article 146. 10.1186/s12916-018-1147-y. 10.1186/s12916-018-1147-y PMC6103973 30131056

[CR85] Triandis HC (2001). Individualism-collectivism and personality. Journal of Personality.

[CR86] Turner, J. C., Hogg, M., Oakes, P., Reicher, S., & Wetherell, M. (1987). *Rediscovering the social group: a self-categorization theory*. Basil Blackwell.

[CR87] The World Bank Group (2022, September 10). Metadata Glossary: Net Migration. https://databank.worldbank.org/metadataglossary/population-estimates-and-projections/series/SM.POP.NETM#:~:text=Net%20migration%20is%20the%20number,for%20the%20five%2Dyear%20period.&text=Net%20migration%20is%20the%20net,including%20both%20citizens%20and%20noncitizens.

[CR88] United States Customs and Immigration Services (2019). Asylum. https://www.uscis.gov/humanitarian/refugees-asylum/asylum.

[CR89] UNHCR (2021, May 10). *Asylum Seekers*. https://www.unhcr.org/en-us/asylum-seekers.html

[CR90] UNHCR (2022a, May 10). What is a Refugee? https://www.unrefugees.org/refugee-facts/what-is-a-refugee/

[CR91] UNHCR (2022b, September 13). *Ukraine Refugee Situation* https://data.unhcr.org/en/situations/ukraine

[CR93] Vaughn MG, Salas-Wright CP, Huang J, Qian Z, Terzis LD, Helton JJ (2017). Adverse childhood experiences among immigrants to the United States. Journal of Interpersonal Violence.

[CR94] Vos SR, Clark-Ginsberg A, Puente-Durán S, Salas-Wright CP, Duque MC, Calderón Herrera IC, Maldonado-Molina MM, Castillo MN, Lee TK, Garcia MF, Fernandez CA, Hanson M, Scaramutti C, Schwartz SJ (2021). The Family Crisis Migration stress Framework: a framework to understand the mental health effects of crisis migration on children and families caused by disasters. New Directions for Child and Adolescent Development.

[CR95] Vranceanu AM, Hobfoll SE, Johnson RJ (2007). Child multi-type maltreatment and associated depression and PTSD symptoms: the role of social support and stress. Child Abuse & Neglect.

[CR96] Wiss, D. A., & Brewerton, T. D. (2020). Adverse childhood experiences and adult obesity: a systematic review of plausible mechanisms and meta-analysis of cross-sectional studies. *Physiology and Behavior*, *223*, Article 112964. 10.1016/j.physbeh.2020.112964. 10.1016/j.physbeh.2020.112964 32479804

[CR97] Wolfe, G. (2021, January 4). *Where are Venezuelan migrants and refugees going? An analysis of legal and social contexts in receiving countries*. Center for Migration Studies. https://cmsny.org/publications/venezuelan-migrants-legal-contexts-wolfe-010421/

[CR98] Xie X, Xia Y, Zhou Z (2004). Strengths and challenges in chinese immigrant families. Great Plains Research.

[CR99] Yoon E, Cabirou L, Galvin S, Hill L, Daskalova P, Bhang C, Baltazar B (2020). A meta-analysis of acculturation and enculturation: Bilinear, multidimensional, and context-dependent processes. The Counseling Psychologist.

[CR100] Yoon E, Lee RM, Goh M (2008). Acculturation, social connectedness, and subjective well-being. Cultural Diversity and Ethnic Minority Psychology.

[CR101] Yoshikawa H, Suárez-Orozco C, Gonzales RG (2017). Unauthorized status and youth development in the United States: Consensus statement of the Society for Research on Adolescence. Journal of Research on Adolescence.

[CR102] Zetino, Y. L., Galicia, B. E., & Venta, A. (2020). Adverse childhood experiences, resilience, and emotional problems in latinx immigrant youth. *Psychiatry Research*, *293*, Article 113450. 10.1016/j.psychres.2020.113450. 10.1016/j.psychres.2020.113450 32977052

